# Seasonal influenza vaccination: Attitudes and practices of healthcare providers in Jordan

**DOI:** 10.1371/journal.pone.0314224

**Published:** 2024-11-21

**Authors:** Olla Hamdan, Justin Z. Amarin, Molly Potter, Zaid Haddadin, Ahmad Yanis, Yanal Shawareb, Najwa Khuri-Bulos, Randa Haddadin, Natasha B. Halasa, Andrew J. Spieker

**Affiliations:** 1 Department of Pediatrics, Division of Pediatric Infectious Diseases, Vanderbilt University Medical Center, Nashville, Tennessee, United States of America; 2 Epidemiology Doctoral Program, School of Medicine, Vanderbilt University, Nashville, Tennessee, United States of America; 3 Department of Pediatrics, School of Medicine, The University of Jordan, Amman, Jordan; 4 Department of Pharmaceutics and Pharmaceutical Technology, School of Pharmacy, The University of Jordan, Amman, Jordan; 5 Department of Biostatistics, Vanderbilt University Medical Center, Nashville, Tennessee, United States of America; Jordan Center For Disease Control (JCDC), JORDAN

## Abstract

**Background:**

Influenza is associated with significant global morbidity and mortality, with vaccination being the primary preventive strategy. Despite recommendations, influenza vaccine uptake among healthcare providers (HCPs) remains suboptimal, especially in the Eastern Mediterranean. We aimed to assess the attitudes and practices of HCPs in Jordan regarding seasonal influenza vaccination and assess sources of variation thereof.

**Methods:**

We conducted a cross-sectional survey study among a sample of HCPs practicing in Jordan (12/29/2020–04/26/2021). Participants completed a questionnaire assessing demographics, influenza vaccination history, attitudes, and practices. We used logistic regression to evaluate factors related to vaccine receipt and reasons for non-vaccination. We used proportional odds models to evaluate factors related to HCP recommendations and to compare opinions on influenza vaccination between ever- and never-vaccinated HCPs.

**Results:**

Of 305 survey initiates, 206 HCPs (67.5%) comprised the analytic sample. The median age was 35 years; 61.2% were women, and 43.7% were pharmacists. Over half (52.9%) never received an influenza vaccine; however, older age and self-identifying as a physician were associated with higher odds of having ever received the influenza vaccine. The main reasons for non-vaccination were related to the misassessment of risks and benefits. Prior receipt of influenza vaccination was strongly associated with odds of recommending vaccination (or = 10.5; 95% CI = [5.38–20.3]; *p*<0.001). The COVID-19 pandemic reportedly enhanced influenza vaccine acceptance among 48.5% of HCPs surveyed.

**Conclusions:**

Low influenza vaccine uptake among healthcare providers in Jordan is related to misassessment of risks and benefits. Enhancing attitudes and confidence through tailored education is crucial to overcoming hesitancy and promoting sustained improvements in vaccination attitudes and practices among HCPs in Jordan.

## Introduction

Influenza contributes to an ongoing major global health burden, with approximately one billion cases occurring annually worldwide, leading to 3–5 million severe cases and 290,000–650,000 influenza-related deaths [1]. Influenza is associated with many complications, including pneumonia, hospitalization, and even death; this is particularly true among high-risk populations such as the elderly, young children, pregnant women, and people with underlying medical conditions [2]. Vaccination is the most effective preventive strategy for reducing transmission, mitigating severe disease, and protecting against serious influenza complications. In the United States, influenza vaccination prevented an estimated 7.09 million illnesses, 3.46 million medical visits, 100,000 hospitalizations, and 7,100 deaths during the 2019–20 season [3].

The Advisory Committee on Immunization Practices (ACIP) recommends annual vaccination for everyone 6 months of age or older without contraindications [4]. Furthermore, the ACIP recommends annual influenza vaccination for all healthcare providers (HCPs) who have the potential to expose their patients to infectious diseases to reduce morbidity and mortality among these providers and their patients [4]. According to a report emanating from the 8^th^ Middle East and North Africa Influenza Stakeholders Network Meeting (held in 2018), 14 participating countries (64%) had established policies for seasonal influenza vaccination [5,6]. Of those, five had incorporated influenza vaccination into national immunization programs, and only three had established specific policies for vaccinating HCPs. Notably, Jordan’s action plan included the following component: “*Improve vaccine coverage in HCPs and high-risk groups*.” Despite these recommendations, influenza vaccine uptake among HCPs remains suboptimal transnationally [5,6]. During the 2020–21 season, only 75.9% of 2,391 HCPs in the United States reported receiving the influenza vaccine [7]. Additionally, data on influenza vaccination rates in the Eastern Mediterranean imply that uptake is suboptimal, with the median influenza vaccination coverage among HCPs estimated to be 28.2% [6].

Since 2006, the World Health Organization Regional Office for the Eastern Mediterranean and CDC have been working to improve surveillance systems to understand better the region’s influenza burden, seasonality, and risk factors; and to establish the need for improved vaccine uptake [8]. According to the fourth meeting of the Eastern Mediterranean Acute Respiratory Infection Surveillance Network, 19 countries in the region, including Jordan, have established functional influenza surveillance systems. However, the progress of these surveillance systems has been challenged by the fragility of healthcare systems and the ongoing humanitarian crises in the region [9]. In general, influenza preparedness in the Eastern Mediterranean is insufficient, and additional data are required to guide response plans [10].

HCPs play a pivotal role in the influenza vaccine uptake as their recommendations are key determinants of vaccine receptivity. Lin *et al*. showed that HCP knowledge, attitudes, and practices regarding vaccination directly influenced their recommendations, thereby affecting patient acceptance [11]. In Jordan, the influenza vaccine is not part of the Ministry of Health’s vaccination program, and there is currently no formal national policy regarding influenza vaccination. However, the government does annually circulate national recommendations for the use of influenza vaccines [12]. Influenza vaccine is available to the public through community pharmacies at a cost and free of charge for healthcare workers in the government sector, though it is not mandatory [13]. A previous study in Jordan performed after the 2015–16 influenza season showed that only 51.9% of HCPs stated ever receiving the influenza vaccine in the past 3 years [14]. However, more recent data are lacking in the region. Therefore, we aimed to describe the self-reported influenza vaccination status of HCPs in Jordan as well as their attitudes and practices regarding influenza vaccination. This study provides unique insights into influenza vaccination practices among a diverse range of healthcare providers in Jordan. It examines multiple aspects: the influence of the COVID-19 pandemic on vaccination attitudes, identification of barriers to vaccination, and exploration of factors affecting vaccine acceptance and recommendation practices to contributes to a more comprehensive understanding of vaccination attitudes and practices in Jordan.

## Materials and methods

### Study design, setting, and participants

We conducted a cross-sectional survey study between December 29, 2020, and April 26, 2021, in Jordan. The study included HCPs actively practicing in hospitals, pharmacies, and private clinics in any one of the 12 governorates of Jordan. The HCPs included physicians, pharmacists, nurses, dentists, and laboratory technicians. Participants were initially recruited via nonrandom convenience sampling, followed by snowball sampling where initial participants referred others for the study.

### Ethical considerations

The study protocol was reviewed and approved by the Institutional Review Board (IRB) of The University of Jordan Deanship of Scientific Research (reference number, 19/2020/580). Due to the anonymous nature of this online survey, the IRB waived the requirement for explicit written informed consent. Instead, implicit informed consent was obtained from all participants, at the beginning of the online survey, participants were presented with information detailing the study’s purpose, ensured confidentiality, and informed participants of their right to withdraw by not submitting the questionnaire. Consent was signified by their voluntary participation and full completion of the survey after reading this information.

### Data collection and measures

We developed a self-administered REDCap questionnaire following a multistep process. We first conducted a comprehensive literature review to identify relevant items [2,7,11,15]. Our research team reviewed and refined the initial pool of items, grouping similar items and eliminating redundancies. The questionnaire was initially developed in English and then translated by a bilingual researcher into Arabic, and four other bilingual researchers reviewed and provided feedback. To establish content validity, two content experts evaluated the items for relevance, clarity, and comprehensiveness. To improve the response process, we conducted a pilot study with 12 healthcare workers. Participants in the pilot study were asked to complete the questionnaire and provide feedback on item clarity, relevance, and overall structure. All feedback from the pilot study was considered and incorporated into the final version of the questionnaire. The final version of the questionnaire is available as supporting information (S1 Appendix). The questionnaire was distributed electronically via social media networks to healthcare providers working in Jordan.

The questionnaire comprised five sections and 38 items. The first section collected demographic and workplace characteristics, including age and years of experience (measured continuously), sex (male or female), professional title (physician, pharmacist, nurse, dentist, or laboratory technician), highest degree (middle college, undergraduate, or graduate), workplace (public hospital, private hospital, community pharmacy, outpatient clinic, or other), and governorate (Amman, az-Zarqā’, Irbid, al-Balqā’, or other).

The second section collected information about the influenza vaccination history of the participants, including whether they ever received the influenza vaccine (“Yes”, “No”, or “Not sure”) and whether their institution partially or fully subsidized the vaccine (“Yes”, “No”, or “Not sure”). Those who indicated having received the vaccine at least once were asked if they were vaccinated for the 2019–20 season, the 2020–21 season, both, or neither. Those who indicated that they were never vaccinated were asked about nine preset reasons for non-vaccination, which we grouped under three domains: misunderstanding safety or efficacy, misassessment of risks and benefits, and convenience. “Misunderstanding safety or efficacy” encompassed concerns about post-vaccination illness, vaccine safety concerns, perceived immune weakening, and skepticism about vaccine effectiveness. The “misassessment of risks and benefits” domain included preferences for natural immunity, perceived lack of contact with high-risk populations, and confidence in one’s immune strength as reasons for non-vaccination. Lastly, “convenience” referred to logistic and financial barriers, including time constraints and unwillingness to incur vaccine costs. This tripartite classification aided in our structured understanding of the motivational factors underlying vaccine refusal.

The third section assessed the practices of HCPs toward influenza vaccination, including to whom they used to recommend the influenza vaccine before the coronavirus disease 2019 (COVID-19) pandemic (everyone, special populations, or no one) and whether the COVID-19 pandemic played a role in enhancing their attitudes and practices toward the influenza vaccine (“Yes”, “No”, or “Not sure”). For those who indicated that the COVID-19 pandemic did play a role, we presented a checklist of five potentially contributory information sources from which they could select one or more.

The fourth and fifth sections evaluated the attitudes of HCPs toward influenza vaccination, and here, we measured responses on a 3-point Likert-type scale (“Agree”, “Neutral”, or “Disagree”).

### Statistical methods

Categorical variables were descriptively summarized using absolute and relative frequencies, while continuous variables were summarized using medians and interquartile ranges (IQRs). We fit a multiple logistic regression model with the receipt of the most recent seasonal influenza vaccine as the outcome and age, sex, profession, workplace, and institutionally subsidized vaccine availability as predictors. Next, we fit three logistic regression models to understand reasons for non-vaccination, grouped under three domains: misunderstanding safety or efficacy, misassessment of risks and benefits, and convenience. The predictors in these models were age, sex, profession, and workplace. We further fit a cumulative-logit proportional odds model with the outcome being whether the healthcare provider recommends the vaccine, using age, sex, profession, workplace, and history of seasonal influenza vaccination as predictors. Finally, we used cumulative-logit proportional odds models to analyze the differential opinions of ever- and never-vaccinated HCPs on eight statements regarding influenza vaccination, measured on a 3-point Likert-type scale. To address missing data in the regression analyses, we used multiple imputation by chained equations with M = 100 iterations, as implemented in the “mice” package in R. All statistical analyses were performed using R (version 4.3.2).

## Results

### Study population

Of the 305 respondents who initiated the survey, 206 (67.5%) completed all sections and were eligible for inclusion. The demographics of the full cohort are summarized in Table 1. In brief, the median age of the respondents was 35 years (IQR, 26–47 years). More respondents were women (*n* = 126; 61.2%). The plurality of HCPs were pharmacists (*n* = 90; 43.7%), followed by physicians (*n* = 55; 26.7%), nurses (*n* = 37; 18.0%), dentists (*n* = 17; 8.3%), and laboratory technicians (*n* = 7; 3.4%). Overall, the cohort had a median of 10 years of experience since graduation (IQR, 2–20 years) and the majority worked in Amman governorate (*n* = 163; 79.1%).

**Table 1:** Demographic characteristics of healthcare providers in Jordan, stratified by self-reporting ever or never having received the seasonal influenza vaccine (*N* = 206).

Characteristic	Total, *N* = 206	Never-vaccinated, *n* = 109	Ever-vaccinated, *n* = 97
Age group (years)—*n* (%)			
18–29	77/204 (37.7)	44/107 (41.1)	33 (34.0)
30–49	89/204 (43.6)	49/107 (45.8)	40 (41.2)
≥50	38/204 (18.6)	14/107 (13.1)	24 (24.7)
Female—*n* (%)	126 (61.2)	79 (72.5)	47 (48.5)
Profession—*n* (%)			
Physician	55 (26.7)	17 (15.6)	38 (39.2)
Pharmacist	90 (43.7)	56 (51.4)	34 (35.1)
Nurse	37 (18.0)	24 (22.0)	13 (13.4)
Dentist	17 (8.3)	7 (6.4)	10 (10.3)
Laboratory technician	7 (3.4)	5 (4.6)	2 (2.1)
Years of experience—*n* (%)			
<5	72/201 (35.8)	39/106 (36.8)	33/95 (34.7)
5–9	21/201 (10.4)	12/106 (11.3)	9/95 (9.5)
10–14	24/201 (11.9)	10/106 (9.4)	14/95 (14.7)
≥15	84/201 (41.8)	45/106 (42.5)	39/95 (41.1)
Highest degree—*n* (%)			
Middle college	25 (12.1)	14 (12.8)	11 (11.3)
Undergraduate	153 (74.3)	82 (75.2)	71 (73.2)
Graduate	28 (13.6)	13 (11.9)	15 (15.5)
Workplace—*n* (%)			
Public hospital	121 (58.7)	61 (56.0)	60 (61.9)
Private hospital	42 (20.4)	20 (18.3)	22 (22.7)
Community pharmacy	16 (7.8)	10 (9.2)	6 (6.2)
Outpatient clinic	6 (2.9)	3 (2.8)	3 (3.1)
Other	21 (10.2)	15 (13.8)	6 (6.2)
Governorate—*n* (%)			
Amman	163 (79.1)	88 (80.7)	75 (77.3)
az-Zarqā’	30 (14.6)	14 (12.8)	16 (16.5)
Irbid	6 (2.9)	2 (1.8)	4 (4.1)
al-Balqā’	3 (1.5)	1 (0.9)	2 (2.1)
Other	4 (1.9)	4 (3.7)	0 (0.0)

### History of influenza vaccination

**Fig 1:**
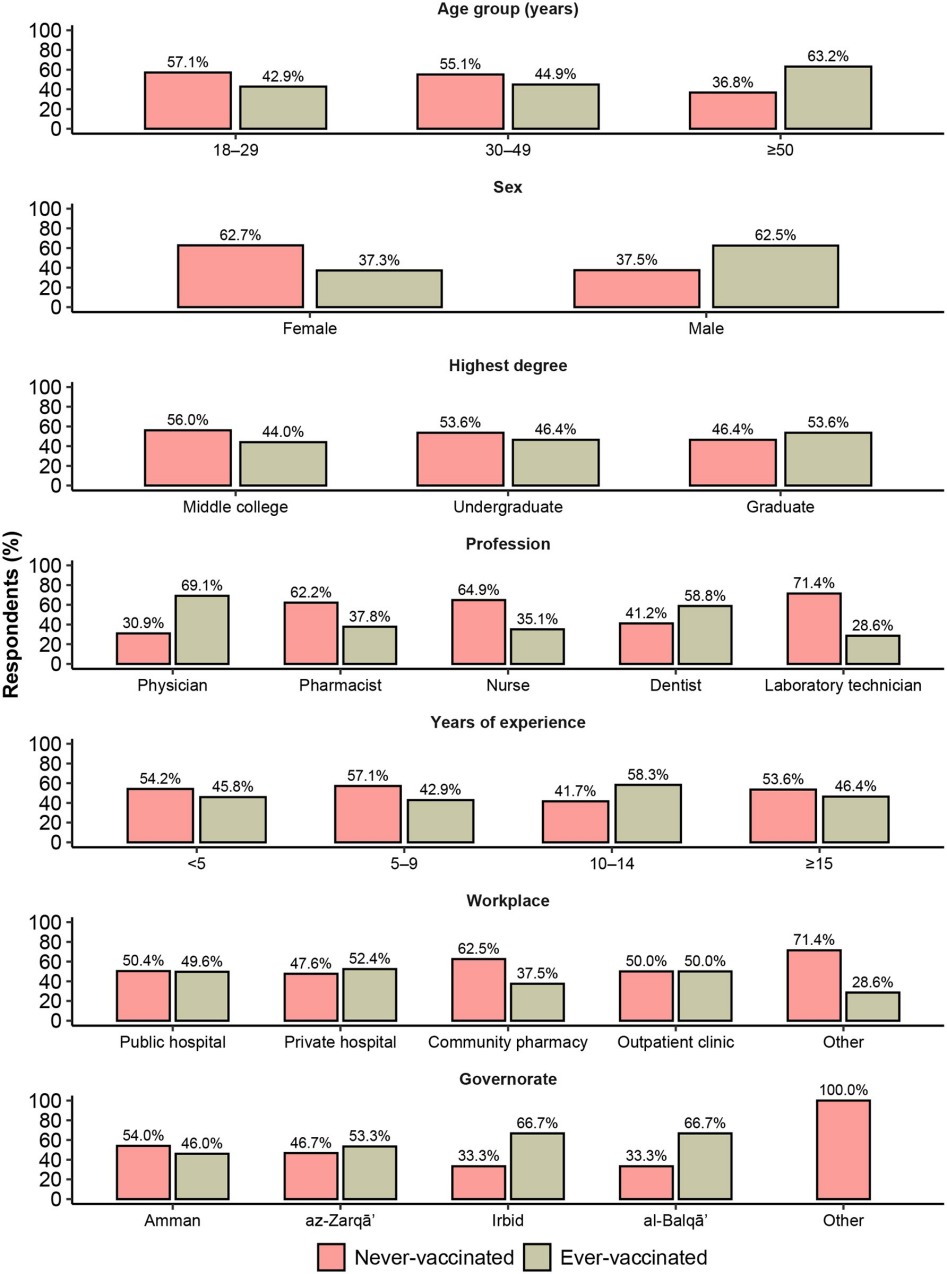
Demographic subgroups of healthcare providers in Jordan, stratified by whether they ever received the seasonal influenza vaccine (*N* = 206).

Overall, 109 respondents (52.9%) never received the seasonal influenza vaccine, while 97 (47.1%) received it at least once. Of those who received an influenza vaccine, 51 (52.6%) received the 2020–21 vaccine, 58 (59.8%) received the 2019–20 vaccine, and 38 (39.2%) received both. The demographics of respondents who did or did not receive the influenza vaccine at least once are compared in Table 1 and Fig 1. Multivariable analysis (Table 2) revealed that older age was associated with higher odds of lifetime history of seasonal influenza vaccination (aOR = 1.04; 95% CI = [1.01–1.07]; *p* = 0.020). In addition, compared with physicians, pharmacists had lower odds of ever receiving an influenza vaccine (aOR = 0.26; 95% CI = [0.11–0.64]; *p* = 0.003) and so did nurses (aOR = 0.26; 95% CI = [0.10–0.68]; *p* = 0.006).

**Table 2:** Predictors of having ever received the seasonal influenza vaccine among healthcare providers in Jordan (*N* = 206).

Predictors	aOR (95% CI)	*p*-value*
Age (years)	1.04 (1.01–1.07)	**0.020**
Male	1.89 (0.98–3.61)	0.056
Profession		
Physician	Reference	
Pharmacist	0.26 (0.11–0.64)	**0.003**
Nurse	0.26 (0.10–0.68)	**0.006**
Other	0.50 (0.17–1.46)	0.21
Workplace		
Public hospital	Reference	
Private hospital	1.50 (0.62–3.61)	0.37
Other	0.82 (0.35–1.96)	0.66
Institutionally subsidized vaccine	1.25 (0.54–2.87)	0.60

*p-values declared statistically significant at a nominal threshold of α = 0.05 are indicated in bold.

### Reasons for non-vaccination

The two most frequently identified reasons for non-vaccination were related to misassessment of risks and benefits (Table 3). Specifically, 36 never-vaccinated respondents (33.0%) selected “*I think my immune system is strong enough to protect me*” as a reason, and 35 (32.1%) selected “*I prefer to get influenza to develop my natural immunity*” as another reason. Compared with physicians, dentists and laboratory technicians as a single group had higher odds of selecting a misassessment-related reason for non-vaccination (aOR = 11.6; 95% CI = [1.68–80.3]; *p* = 0.013), and nurses had lower odds of selecting a convenience-related reason for non-vaccination (aOR = 0.09; 95% CI = [0.01–0.65]; *p* = 0.017). Otherwise, multivariable models did not provide sufficient evidence for an association between age, sex, profession, or workplace and any of the three domains (Table 4).

**Table 3:** Reasons reported by healthcare providers in Jordan for never having received the seasonal influenza vaccine were grouped under three domains (*n* = 109).

Reason	*n* (%)*
Misunderstanding safety or efficacy	
“I or someone I know got sick after taking the influenza vaccine.”	11 (10.1)
“I have concerns regarding the vaccine’s safety.”	16 (14.7)
“Influenza vaccine weakens the immune system.”	4 (3.6)
“I do not believe the influenza vaccine is effective.”	12 (11.1)
Misassessment of risks and benefits	
“I prefer to get influenza in order to develop natural immunity.”	35 (32.1)
“I am not in contact with high-risk individuals.”	5 (4.6)
“I think my immune system is strong enough to protect me.”	36 (33.0)
Convenience	
“I do not have time.”	10 (9.2)
“I do not want to pay for it.”	2 (1.8)
Other	9 (8.3)

*Participants had the option to select multiple answers.

**Table 4:** Predictors of reasons for never having received the seasonal influenza vaccine among healthcare providers in Jordan (*n* = 109).

Predictors	Misunderstanding safety or efficacy		Misassessment of risks and benefits		Convenience	
	aOR (95% CI)	*p*-value*	aOR (95% CI)	*p*-value*	aOR (95% CI)	*p*-value*
Age (years)	0.98 (0.93–1.03)	0.35	1.03 (0.99–1.07)	0.20	0.93 (0.84–1.03)	0.14
Male	1.38 (0.50–3.80)	0.54	1.56 (0.55–4.42)	0.40	0.60 (0.13–2.69)	0.50
Profession^2^						
Physician	Reference		Reference		Reference	
Pharmacist	2.44 (0.48–12.3)	0.28	2.67 (0.70–10.3)	0.15	0.16 (0.03–1.02)	0.053
Nurse	4.16 (0.63–27.4)	0.14	2.42 (0.59–9.90)	0.22	0.09 (0.01–0.65)	**0.017**
Other	0.33 (0.03–3.68)	0.36	11.6 (1.68–80.3)	**0.013**	NA^2^	
Workplace						
Public hospital	Reference		Reference		Reference	
Private hospital	0.48 (0.09–2.63)	0.40	0.79 (0.23–2.72)	0.70	3.12 (0.51–19.2)	0.22
Other	0.65 (0.23–1.83)	0.41	1.29 (0.44–3.81)	0.64	2.02 (0.23–17.5)	0.52

*p-values declared statistically significant at a nominal threshold of α = 0.05 are indicated in bold.

^2^None of the healthcare providers in the “Other” category reported a convenience-related reason for non-vaccination.

### Recommending vaccination

At the time of the survey, reflecting on their practices before the COVID-19 pandemic, 58 respondents (28.2%) indicated that they never recommended the influenza vaccine, 76 (36.9%) recommended it to special populations (pregnant women, children of 6 months or older, individuals 65 years or older, and individuals with chronic diseases), and 72 (35.0%) recommended it to everyone. By profession, 10.9% of physicians (*n* = 6) indicated that they never recommended the influenza vaccine, while 25.6% of pharmacists (*n* = 23), 48.6% of nurses (*n* = 18), and 45.8% of other HCPs (*n* = 11) indicated the same. We did not identify sufficient evidence of an association between age, sex, specific profession, or workplace and the odds of recommending vaccination (Table 5). However, a history of influenza vaccination was strongly associated with odds of recommending vaccination (aOR = 10.5; 95% CI = [5.38–20.3]; *p*<0.001).

**Table 5:** Predictors of recommending the seasonal influenza vaccine among healthcare providers in Jordan (*N* = 206).

Predictors	aOR (95% CI)	*p*-value*
Age (years)	1.01 (0.98–1.04)	0.42
Male	0.89 (0.47–1.67)	0.72
Profession		
Physician	Reference	
Pharmacist	0.69 (0.31–1.54)	0.36
Nurse	0.39 (0.14–1.10)	0.075
Other	0.34 (0.12–0.96)	**0.042**
Workplace		
Public hospital	Reference	
Private hospital	2.19 (0.91–4.79)	0.081
Other	2.30 (1.10–4.79)	**0.027**
History of seasonal influenza vaccination	10.5 (5.38–20.3)	**<0.001**

*p-values declared statistically significant at a nominal threshold of α = 0.05 are indicated in bold.

### Opinions on influenza vaccination

**Fig 2:**
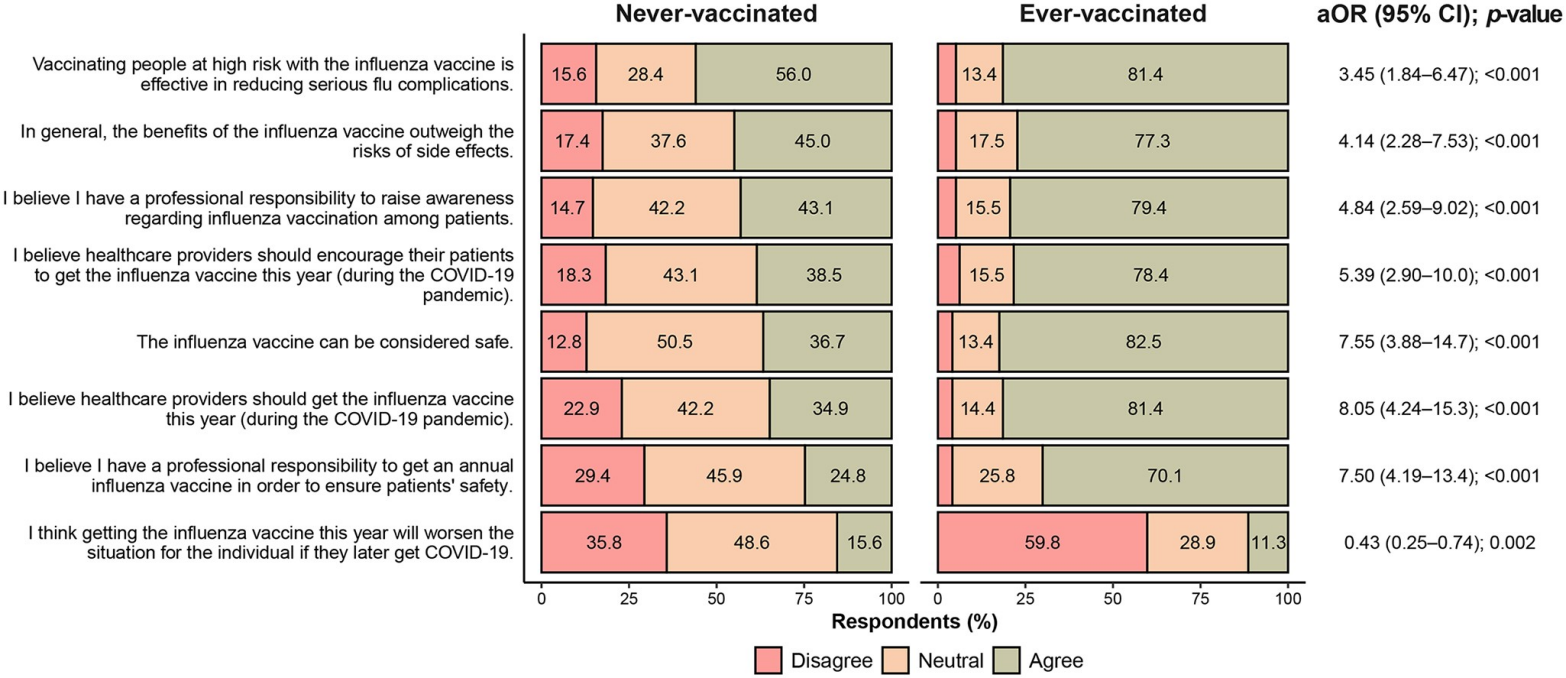
Opinions of ever- and never-vaccinated healthcare providers in Jordan on eight statements regarding influenza vaccination. Adjusted odds ratios (aORs) were estimated using cumulative-logit proportional odds models (*N* = 206).

We asked respondents to judge a set of eight statements using a 3-point Likert scale. Fig 2 shows the distribution of responses stratified by vaccination status. The distribution of responses differed between ever- and never-vaccinated HCPs for all statements presented. We also asked HCPs about reasons for non-vaccination among the general public in Jordan: 116/194 (59.8%) agreed “they are scared of experiencing severe side effects after influenza vaccine,” 126 (61.2%) agreed “they are unaware of the benefits of influenza vaccine,” 136 (66.0%) agreed “they do not believe in influenza vaccination and prefer natural immunity after influenza infection,” 145 (70.4%) agreed “they have safety concerns about its use,” 89 (43.2%) agreed “the cost of influenza vaccine is not covered by insurance,” 73 (35.4%) agreed “they are unaware of its availability or where to get it,” and 113 (54.9%) agreed “they believe they are healthy and do not need the influenza vaccine.” Overall, 100 HCPs (48.5%) reported that the COVID-19 pandemic played a role in enhancing their attitudes and practices toward the influenza vaccine. Among those 100, the most common factor that affected their attitudes and practices was fear of the extra burden of influenza on the healthcare system (*n* = 57; 57.0%), followed by reading scientific reports and journal articles (*n* = 54; 54.0%), information available on websites and social media (*n* = 23; 23.0%), institutional lectures or brochures (*n* = 21; 21.0%), and information available on TV and in newspapers (*n* = 12; 12.0%).

## Discussion

We studied the attitudes and practices of a convenience sample of HCPs in Jordan regarding influenza vaccination. Our study showed several key findings. First, nearly half of the HCPs reported ever receiving the influenza vaccine. Second, misassessment of the risks and benefits was the main reported domain for suboptimal influenza vaccine uptake among HCPs. Third, ever-vaccinated HCPs tended to have more positive attitudes about the vaccine. Fourth, the COVID-19 pandemic positively influenced attitudes and practices toward influenza vaccination.

Our findings align with broader regional trends in influenza vaccination coverage among HCPs. The low influenza vaccination coverage observed in our study is consistent with the low median influenza vaccination coverage among HCPs in the Eastern Mediterranean region (28.2%) as reported by Zaraket *et al*. [6]. A previous study in Jordan performed after the 2015–16 influenza season showed that only 51.9% of HCPs stated ever receiving the influenza vaccine in the past 3 years, and only 30.8% reported receiving the influenza vaccine in the 2015–16 season [14]. This persistent low coverage suggests that significant barriers to vaccination remain present, highlighting the need for targeted interventions to improve vaccine uptake among HCPs.

We observed disparities in vaccination coverage across different healthcare professions, consistent with existing literature [16,17]. Our study found higher vaccination coverage among physicians compared to other HCPs. Other studies have attributed the lower vaccination coverage among nurses to higher vaccine hesitancy, influenced by several factors such as the perceived risks and doubts about vaccine effectiveness, alongside concerns about side effects like injection pain. Mistaken immunologic beliefs, preference for non-vaccine prevention methods, and challenges in healthcare access also play roles [18]. These profession-specific barriers highlight the need for tailored interventions. Crucial steps may include implementing educational programs and embedding vaccine advocacy in professional training. Additionally, policy interventions such as mandating influenza vaccination among HCPs have shown promise in other contexts, potentially increasing vaccination coverage to over 90% when coupled with comprehensive campaigns and consequences for noncompliance [19].

Our study showed important insights into the reasons for vaccine hesitancy among HCPs. Among never-vaccinated HCPs, the most reported reasons for non-vaccination were related to misassessment of the risk-benefit ratio associated with the vaccine. This finding aligns with a previous study conducted in Jordan, in which never-vaccinated HCPs also expressed skepticism about the benefits of seasonal influenza vaccination. Furthermore, significant differences in health beliefs were observed between HCPs who intended to receive the vaccine versus those who did not [20]. Our results diverge from some previous research regarding barriers to vaccination. Only a minority of participants identified time and financial constraints as barriers to vaccination suggesting that in our sample access to and affordability of the vaccine are not the primary obstacles. This contrasts with findings in other countries that showed time as a major obstacle to vaccination [14,21]. Understanding and addressing the rationale behind vaccine hesitancy among HCPs may significantly contribute to overcoming the existing challenges of low vaccination coverage among HCPs.

Healthcare providers believed that safety concerns associated with the vaccine were the primary barriers against vaccination among the general public in Jordan, which supports the results of a previous survey study of 941 adults in Jordan that identified safety and efficacy concerns as the key barriers to influenza vaccine acceptance [22]. The impact of the COVID-19 pandemic on attitudes toward influenza vaccination emerged as a significant finding in our study. Nearly half of the HCPs reported that the COVID-19 pandemic played a positive role in enhancing their attitudes and practices toward the influenza vaccine. This is consistent with a recent study of 1,218 HCPs in Jordan regarding influenza vaccination, which indicated that 63% of HCPs reported receiving the influenza vaccine during the 2021–22 influenza season, possibly attributed to the increased awareness of vaccination importance during the COVID-19 pandemic [17]. However, the translation of this increased awareness into sustained behavior change remains uncertain, a study of a sample of Jordanian adults during the COVID-19 pandemic showed that 804 of 1,112 respondents (72.3%) were not willing to take the influenza vaccine. Regarding influenza vaccine education, only 10.3% of respondents identified physicians as a source of information [23]. These findings highlight a significant gap in public vaccine education and suggest an urgent need for effective communication strategies, particularly from HCPs, to address the concerns of their patients and improve influenza vaccine uptake in Jordan.

Ever-vaccinated HCPs were more confident about the vaccine’s safety, efficacy, and benefits and were more likely to recognize their professional responsibility in promoting vaccination compared to never-vaccinated HCPs. This could be due to several factors, including time constraints, heavy workload, inadequate resources, and HCPs’ lack of confidence in having sufficient and updated information to address parents’ questions and concerns regarding vaccination. Paterson *et al*. showed that the HCPs’ recommendations are the strongest and most important factor in promoting vaccination among their patients [24]. In a previous survey conducted among adults in Jordan, the primary driver of vaccine acceptance among vaccinated participants was the recommendations provided by their HCPs; most participants were more willing to be vaccinated if it was recommended by an HCP [22]. In a survey of a sample from the Jordanian population, 77.1% of respondents said they would get vaccinated if advised by a physician, as opposed to making a self-determined decision [15]. Our findings, coupled with those of prior studies, indicate that there is a pressing need for educational campaigns about influenza vaccination specifically targeting HCPs in Jordan [25].

Our study has several limitations. First, due to the constraints imposed by the COVID-19 pandemic, the survey was conducted online, restricting our sample to HCPs with access to online platforms. Second, our sample predominantly consisted of physicians and pharmacists residing in Amman and was relatively small. Therefore, the results may not be generalizable to the broader healthcare workforce in Jordan. However, the distribution of key demographic characteristics was relatively similar to previously reported statistics on the population of interest [26]. In Jordan, as of 2016, the distribution of the national health workforce by profession was as follows: 26% physicians, 30% registered nurses, 28% pharmacists, 12% dentists and 4% midwives [26]. Moreover, Amman has a considerably larger healthcare workforce density than other governorates; for example, Amman has 19.6 physicians and 22.4 nurses per 10,000 population, while az-Zarqā’ has 6.9 of each per 10,000 population [27]. In addition, in 2013, women comprised 44% of HCPs in Jordan, and about 85% of all HCPs were under 50 years old with 40% being 30 years old or younger [28]. This alignment of key demographic variables suggests that, while not necessarily representative, our findings may provide valuable insights into the experiences and perspectives of a significant portion of HCPs in Jordan. Another limitation of our study is that the survey data is self-reported, which could lead to socially desirable responding. While we aimed to mitigate this limitation by administering the survey anonymously, the absence of a comprehensive national record of influenza vaccination in Jordan makes this a persistent issue. To maintain participant anonymity and reach a diverse range of providers, we did not collect data on specific healthcare facilities or their exact number. Instead, we gathered information on the type of workplace (e.g., governmental, private) from each participant. Also, we employed a combination of convenience and snowball sampling techniques, which may have introduced selection bias. These non-probability sampling methods, while practical for our study context, may have led to overrepresentation of certain HCP groups or geographic areas which can limit the generalizability of our findings to the broader HCP population in Jordan. Additionally, we initially considered including years of experience as a predictor in our models, we found it to be highly correlated with age in our dataset, potentially leading to multicollinearity issues in our analysis. Despite these limitations, our study provides valuable insights into the vaccination attitudes of HCPs in Jordan, serving as an important foundation for future, more comprehensive studies.

In conclusion, our study highlights critical gaps in attitudes and practices toward the influenza vaccine among a sample of HCPs in Jordan. Addressing these gaps is crucial for enhancing vaccination coverage. Tailored educational and awareness initiatives can play a pivotal role in clarifying misconceptions about the vaccine’s risks and benefits. Furthermore, underscoring the professional duty of HCPs to both receive and advocate for annual vaccinations could be influential. The heightened acceptance of influenza vaccination during the COVID-19 pandemic presents a unique opportunity for sustained messaging on the value of influenza immunization. Addressing hesitancy and mandating vaccination among HCPs may foster broader vaccine adoption due to strengthened provider endorsements to their patients.

## Supporting information

S1 Appendix Survey questionnaire on seasonal influenza vaccination attitudes and practices among healthcare providers in Jordan (12/29/2020–04/26/2021). (PDF)

S1 File (XLSX)
