# Supplementary material for: Seasonal influenza vaccination: Attitudes and practices of healthcare providers in Jordan
Source: PLoS One. 2024 Nov 21;19(11):e0314224. doi: 10.1371/journal.pone.0314224 (PMC11581314; doi:10.1371/journal.pone.0314224)
Supplement: S1 Appendix — (PDF) [file pone.0314224.s001.pdf]

THE QUESTIONNAIRE

Seasonal influenza vaccination: attitude, practices, and barriers among health care providers in Jordan before and after COVID-19 pandemic.

This study is conducted by a group of researchers from The University of Jordan and Vanderbilt University/USA. This study aims to evaluate the attitudes, practices, and barriers of health care providers in Jordan towards seasonal influenza vaccines, and also to assess how COVID-19 pandemic affected the practices and perceptions of health care providers towards influenza vaccines. The information provided will be treated in strict confidentiality for scientific purpose only. You will never be asked to reveal your identity.
Filling the questionnaire and submitting your answers mean that you are consenting to participate in the study. You can at any time withdraw from the study by not submitting the questionnaire.
The survey has 5 sections and takes 4-8 minutes to be completed. Thank you for your participation!

يقوم بهذه الدراسه عدد من الباحثين في الجامعة الاردنية ,جامعة ڤاندربيلت/أمريكا. تهدف هذه الدراسة إلى تقييم المواقف والممارسات والموانع لدى مقدمي الرعاية الصحية في الأردن تجاه لقاحات الأنفلونزا الموسمية، وتقييم أثر جائحة كوفيد-19 على ممارسات مقدمي الرعاية الصحية تجاه لقاحات الأنفلونزا الموسمية. سيتم التعامل مع المعلومات المقدمة بسرية تامة للأغراض العلمية فقط. ولن يُطلب منك أبدًا الكشف عن هويتك.
ملء الاستبيان وتقديم إجاباتك يعني أنك موافق على المشاركة في الدراسة. يمكنك الانسحاب من الدراسة في أي وقت من خلال عدم تسليم الاستبيان.
يتكون الإستبيان من 5 أقسام ويحتاج من 4 إلى 8 دقائق لإكماله. شكرا على مشاركتك !

SECTION ONE

Gender الجنس ☐ male ذكر ☐ female أنثى

Age العمر ________

Professional title المسمى الوظيفي : ☐ Physician, طبيب ☐ Dentist, طبيب اسنان ☐ Nurse, ممرض
☐ Pharmacist, صيدلاني ☐ Clinical pharmacist, صيدلي سريري ☐ Laboratory technician, فني مختبرات طبي

specify specialty (if applicable) التخصص _________

Years of experience since graduation, عدد سنوات الخبرة منذ التخرج: ________

Qualification/degree, الدرجة العلمية: ☐ Middle college ,كليه ☐ University degree ,بكالوريوس
☐ MSc or PhD دكتوراه , ماجستير

Workplace مكان العمل ☐ Public hospital, مستشفى حكومي ☐ Private hospital, مستشفى خاص
☐ Community pharmacy, صيدلية مجتمع ☐ Outpatient's clinic, عيادة خاصة ☐ Private Laboratory, مختبر خاص
☐ Others, please specify أخرى، يرجى التحديد ________

Location of your institution (city, zone), موقع مؤسسة العمل: ☐ Amman, عمان ☐ Irbid, إربد
☐ Zarqa, الزرقاء ☐ Ajloun, عجلون ☐ Jarash, جرش ☐ Balqa, البلقاء ☐ Mafraq, المفرق
☐ Madaba, مأدبا ☐ karak, الكرك ☐ Tafila, الطفيله ☐ Ma'an, معان ☐ Aqaba, العقبه

SECTION TWO

- **1) Have you ever received the influenza vaccine?**هل تلقيت لقاح الإنفلونزا من قبل

  ☐ Yes, نعم ☐ No,لا ☐ Not sure, غير متأكد

  If your answer was yes, go to question 2, لو أجبت بنعم يرجى الانتقال الى سؤال 2
  If your answer was no, go to question 3, لو أجبت بلا يرجى الانتقال الى سؤال 3

- **2) Did you get the previous influenza vaccine between (September 2019- March 2020)?**
  هل تلقيت لقاح الانفلونزا السابق بين (سبتمبر 2019 - مارس 2020)
  ☐ Yes, نعم ☐ No,لا ☐ Not sure, غير متأكد

  Did you get the current influenza vaccine (between September 2020-up till now?
  هل تلقيت لقاح الانفلونزا الحالي بين (سبتمبر 2020 – حتى الان)
  ☐ Yes, نعم ☐ No,لا ☐ Not sure, غير متأكد

- **3) If your answer was no, why?** لا، لماذا *Check all that apply*

  ☐ I do not have time, لا املك الوقت
  ☐ I or someone I know got sick after taking the influenza vaccine لقد مرضت أنا / شخص أعرفه بعد أخذ لقاح الانفلونزا سابقا
  ☐ I have concerns regarding the vaccine's safety, لدي مخاوف بشأن مأمونية لقاح الانفلونزا
  ☐ Influenza vaccine weakens the immune system, لقاح الانفلونزا يضعف جهاز المناعة
  ☐ I do not believe the influenza vaccine is effective, انا لا اؤمن بفاعلية لقاح الانفلونزا
  ☐ I prefer to get influenza in order to develop natural immunity, أفضل الاصابه بالانلفونزا من أجل تطوير مناعة طبيعية للفايروس
  ☐ I am not in contact with high risk individuals, انا لست على اتصال مباشر بأفراد معرضين لمخاطر عالية من الانفلونزا
  ☐ I think my immune system is strong enough to protect me, اعتقد ان جهازي المناعي قوي بشكل كاف لحمايتي
  ☐ I do not want to pay for it, لا أريد ان أتحمل تكلفته
  ☐ others, اخرى____

- **Does your institution provide you with influenza vaccine free of charge or with reduced charge?**هل تزودك مؤسستك بلقاح الإنفلونزا مجانًا أو بتكلفة مخفضة؟
  ☐ Yes, with reduced price نعم بسعر مخفض ☐ Yes, free of charge, نعم ، مجاناً
  ☐ No لا ☐ Not sure غير متأكد

- **This influenza season (2020-2021),during COVID-19 pandemic, Did you get/ planning to get the influenza vaccine.** في موسم الانفلونزا الحالي (2021/2020), خلال جائحة فايروس كورونا المستجد,هل تلقيت أو تخطط لتلقي لقاح الانفلونزا؟
  ☐ Yes, نعم ☐ No,لا ☐ Not sure, غير متأكد

SECTION THREE

Please answer the below questions based on your **actual practices.**

- **In the past (before COVID-19 pandemic) during my practice, I used to recommend the influenza vaccine for:** في الماضي (قبل جائحة فايروس كورونا المستجد) أثناء ممارستي ، كنت أوصي بلقاح الإنفلونزا من أجل

  ☐ Everyone, الجميع ☐ Only some people/special population, فئة معينة

  ☐ Never recommended لم أوصي به

- If your answer was "Only special population/ the special population" that you used to recommend the influenza vaccine for includes which of these categories? لو كانت اجابتك فئة معينة فإن هذه الفئة التي كنت توصيها بأخذ لقاح الانفلونزا تشمل اي من هذه الفئات

  ☐ pregnant women, النساء الحوامل ☐ children of 6 months or older, الاطفال من سن 6 اشهر او اكبر

  ☐ people 65 years or older, الاشخاص 65 سنة او اكبر

  ☐ individuals with chronic diseases,المصابين بأمراض مزمنة ☐ all of the above, جميع ما ذكر أعلاه

  ☐ others آخرون____________

- **This year (during COVID-19 pandemic) in my practice, I recommend the influenza vaccine for:** هذه السنة (خلال جائحة فايروس كورونا المستجد) أثناء ممارستي ، كنت أوصي بلقاح الإنفلونزا من أجل:

  ☐ Everyone who doesn't have contraindication, الجميع، ما لم يكن لديهم موانع طبية للإستخدام

  ☐ Only some people / special population, فئة معينة

  ☐ I don't recommend it لا أوصي به

- If your answer was "Only special population/ the special population" that you used to recommend the influenza vaccine for includes which of these categories? لو كانت اجابتك فئة معينة فإن هذه الفئة التي كنت توصيها بأخذ لقاح الانفلونزا تشمل اي من هذه الفئات

  ☐ pregnant women, النساء الحوامل ☐ children of 6 months or older, الاطفال من سن 6 اشهر او اكبر

  ☐ people 65 years or older, الاشخاص 65 سنة او اكبر

  ☐ individuals with chronic diseases,المصابين بأمراض مزمنة ☐ all of the above, جميع ما ذكر أعلاه

  ☐ others آخرون____________

- **In general, I always recommend the influenza vaccine to all my family members/ friends.** بشكل عام، أوصي دائمًا بلقاح الإنفلونزا لجميع أفراد عائلتي / أصدقائي

  ☐ yes, if they don't have contraindications نعم، طالما ليس هناك موانع طبية للإستخدام

  ☐ No لا ☐ Not sure غير متأكد

- **COVID-19 pandemic played a role in enhancing my practices and attitudes towards the influenza vaccine If you answer No or Not sure, please go to the next section** لعبت جائحة فايروس كورونا المستجد دورًا في تعزيز ممارساتي ومواقفي تجاه لقاح الإنفلونزا، لو أجبت بلا او غير متأكد،فيرجى الانتقال الى القسم التالي

  ☐ Yes, نعم ☐ No,لا ☐ Not sure, غير متأكد

- **Since you answered Yes to the previous question, which of the following played a role in improving your acceptance to influenza vaccine** بما أنك أجبت بنعم على السؤال السابق، أي من الآتي ساهم في زيادة قبولك للقاح الإنفلونزا
☐ Information available in TV and newspapers, المعلومات المتوفرة في التلفزيون والصحف
☐ Information available in websites and social media, المعلومات المتوفرة على المواقع الالكترونية و مواقع التواصل الاجتماعي
☐ Reading scientific reports and journal articles, قراءة التقارير و البحوث في المجلات العلمية
☐ Lectures and /or brochures from my institution, محاضرات و حملات توعوية من مؤسسة عمل
☐ Fear of the extra burden influenza can cause on the health care system, الخوف من العبء الاضافي الذي يمكن ان تسببه الانفلونزا على النظام الصحي

- **This year, I have noticed that COVID-19 increased influenza vaccine acceptance and more people are taking the influenza vaccine compared to previous years .** لاحظت هذا العام أن جائحة فايروس كورونا، زادت من قبول لقاح الإنفلونزا وأن المزيد من الناس يأخذون اللقاح مقارنة بالسنوات السابقة
☐ Yes, نعم ☐ No,لا ☐ Not sure, غير متأكد

- **In general, I follow up the guidelines published by the Advisory Committee on Immunization Practices (ACIP) or Centre for Disease Control (CDC-USA) regarding influenza immunization.** بشكل عام ، أنا أتابع الإرشادات التي نشرتها اللجنة الاستشارية لممارسات التحصين أو مركز السيطرة على الأمراض الأمريكي فيما يتعلق بلقاح الإنفلونزا
☐ Yes, نعم ☐ No,لا ☐ Not sure, غير متأكد ☐ Never heard about it, لم اسمع بها

SECTION FOUR

Please answer the below questions based on your own opinions
الرجاء الإجابة على الأسئلة التالية بناءً على آرائك الشخصية

- **Vaccinating people at high risk with influenza vaccine is effective in reducing serious flu complications** تطعيم الأشخاص ذوي الاختطار الكبير بمطعوم الانفلونزا سوف يقلل من خطورة المضاعفات التي يمكن ان تصيبهم
☐ Agree أوافق ☐ Neutral محايد ☐ Disagree لا أوافق
- **In general, the benefits of influenza vaccine outweigh the risks of side effects** بشكل عام ، فوائد لقاح الأنفلونزا تفوق مخاطر آثاره الجانبية
☐ Agree أوافق ☐ Neutral محايد ☐ Disagree لا أوافق
- **Influenza vaccine can be considered safe** يمكن اعتبار لقاح الإنفلونزا آمنًا
☐ Agree أوافق ☐ Neutral محايد ☐ Disagree لا أوافق
- **I believe I have a professional responsibility to get an annual influenza vaccine in order to ensure patient's safety** أؤمن أن لدي مسؤولية مهنية للحصول على لقاح الإنفلونزا سنويا من أجل ضمان سلامة المرضى
☐ Agree أوافق ☐ Neutral محايد ☐ Disagree لا أوافق
- **I believe I have a professional responsibility to raise awareness regarding influenza vaccination to patients** أؤمن أن لدي مسؤولية مهنية لرفع مستوى الوعي لدى المرضى بشأن التطعيم ضد الإنفلونزا
☐ Agree أوافق ☐ Neutral محايد ☐ Disagree لا أوافق

- **I think getting the influenza vaccine this year will worsen the situation of the individual if later on gets COVID-19** أعتقد أن التطعيم بلقاح الإنفلونزا هذا العام سيزيد من سوء حالة الفرد إذا أصيب لاحقًا بفايروس كورونا
  ☐ Agree أوافق ☐ Neutral محايد ☐ Disagree لا أوافق
- **I believe healthcare providers should get influenza vaccine this year (during COVID-19 pandemic)** أعتقد أن مقدمي الرعاية الصحية يجب أن يحصلوا على لقاح الأنفلونزا هذا العام خلال جائحة فايروس كورونا المستجد
  ☐ Agree أوافق ☐ Neutral محايد ☐ Disagree لا أوافق
- **I believe healthcare providers should encourage their patients to get influenza vaccine this year (during COVID-19 pandemic)** أعتقد أنه يجب على مقدمي الرعاية الصحية تشجيع مرضاهم على الحصول على لقاح الأنفلونزا هذا العام خلال جائحة فايروس كورونا المستجد
  ☐ Agree أوافق ☐ Neutral محايد ☐ Disagree لا أوافق

SECTION FIVE

In your opinion, people in Jordan do not get the influenza vaccine because
برأيك الاردنيون لا يتلقون لقاح الأنفلونزا للأسباب التالية

- **They are scared of experiencing severe side effects after influenza vaccine,** الخوف من الإصابة بأعراض جانبية شديدة بعد تلقي لقاح الأنفلونزا
  ☐ Agree أوافق ☐ Neutral محايد ☐ Disagree لا أوافق
- **They are unaware of the benefits of influenza vaccine,** قلة الوعي بفوائد تلقي لقاح الانفلونزا
  ☐ Agree أوافق ☐ Neutral محايد ☐ Disagree لا أوافق
- **They do not believe in influenza vaccination and prefer natural immunity after influenza infection,** لا يؤمنون بالتطعيم ضد الإنفلونزا ويفضلون المناعة الطبيعية بعد الإصابة بالأنفلونزا
  ☐ Agree أوافق ☐ Neutral محايد ☐ Disagree لا أوافق
- **They have safety concerns about its use,** لديهم مخاوف تتعلق بمأمونية استخدام لقاح الانفلونزا
  ☐ Agree أوافق ☐ Neutral محايد ☐ Disagree لا أوافق
- **The cost of influenza vaccine is not covered by insurance,** لا يغطي التأمين تكلفة لقاح الأنفلونزا
  ☐ Agree أوافق ☐ Neutral محايد ☐ Disagree لا أوافق
- **They are unaware of its availability or where to get it,** غير مدركين لتوافر المطعوم أو طرق الحصول عليه
  ☐ Agree أوافق ☐ Neutral محايد ☐ Disagree لا أوافق
- **They believe they are healthy and do not need the influenza vaccine,** يعتقدون أنهم يتمتعون بصحة جيدة ولا يحتاجون إلى لقاح الأنفلونزا
  ☐ Agree أوافق ☐ Neutral محايد ☐ Disagree لا أوافق
